# Strain structure analysis of *Mycobacterium tuberculosis* circulating among HIV negative, positive and drug resistant TB patients attending chest clinics in Western Kenya

**DOI:** 10.1186/s12890-023-02802-z

**Published:** 2023-12-09

**Authors:** Martin O. Ogwang, Lameck Diero, Florence Ng’ong’a, Gabriel Magoma, Lucy Mutharia, Mabel Imbuga, Caroline Ngugi

**Affiliations:** 1 School of Public Health Nairobi Kenya, Jomo Kenyatta University of Agriculture and Technology, Juja, Kenya; 2 School of Medicine, Moi University, Eldoret, Kenya; 3 School of Biomedical Sciences, Jomo Kenyatta University of Agriculture and Technology, Nairobi, Kenya; 4 Department of Cellular and Molecular Biology, University of Guelph, Guelph, ON Canada

**Keywords:** Spoligotyping, Tuberculosis epidemiology, Strain structure, Drug-resistance

## Abstract

**Background:**

Despite global tuberculosis (TB) interventions, the disease remains one of the major public health concerns. Kenya is ranked 15th among 22 high burden TB countries globally.

**Methods:**

A cross-sectional study was conducted in Western Kenya, which comprises 10 counties. A multistage sampling method was used where a single sub-county was randomly selected followed by sampling two high volume health facility from each sub-county. Identification of spoligotype profiles and their family distribution and lineage level were achieved by comparison with SITVIT database.

**Results:**

Lineage distribution pattern revealed that the most predominant lineage was CAS 220 (39.8%) followed by Beijing 128 (23.1%). The other lineages identified were T, LAM, H, X, S and MANU which were quantified as 87 (15.7%), 67 (12.1%), 16 (2.8%), 10 (1.8%), 8 (1.4%) and 5 (0.9%) respectively. CAS and Beijing strains were the most predominant lineage in both HIV negative and positive TB patients. The Beijing lineage was also the most predominant in resistant *M. tuberculosis* strains as compared to wild type. A total of 12 (2.0%) were orphaned *M. tuberculosis* strains which were spread across all the 10 counties of the study site. In multivariate logistic regression adjusting for potential cofounders three potential risk factors were significant. HIV status (OR = 1.52, CI = 0.29–3.68 and *P* value of 0.001), Alcohol use (OR = 0.59, CI = 0.43–3.12 and *P*-value =0.001) and cross border travel (OR = 0.61, CI = 0.49–3.87 and *P* value = 0.026). Most *M. tuberculosis* clinical isolates showed genetic clustering with multivariate logistic regression indicating three potential risk factors to clustering. HIV status (OR = 1.52, CI = 0.29–3.68 and P value of 0.001), Alcohol use (OR = 0.59, CI = 0.43–3.12 and P-value =0.001) and cross border travel (OR = 0.61, CI = 0.49–3.87 and P value = 0.026).

**Conclusion:**

There exist diverse strains of *M. tuberculosis* across the 10 counties of Western Kenya. Predominant distribution of clustered genotype points to the fact that most TB cases in this region are as a result of resent transmission other than activation of latent TB.

## Introduction

Tuberculosis remains a global threat to public health and is the leading cause of death by a single infectious agent with 1.6 million deaths in 2017. An estimated 10 million people developed TB disease in 2017 but only 6.4 million (61%) were notified [1]. Despite the global aim of a 95% reduction in TB deaths, 90% reduction in incidence and 0% TB affected families facing catastrophic costs due to TB by 2035 [2], the disease still remains a major challenge on livelihood. The emergence of resistant strains to first- and second-line treatment regimen has seen an upsurge of monoresistant, multidrug resistant and even pre-extensive multidrug resistant tuberculosis. Surprisingly, *Mycobacterium tuberculosis,* the causative agent of tuberculosis, is capable of surviving and replicating within macrophages and other human cells.

For this reason, the bacterium has developed a unique mechanism to gain entry and survive in host cells. With the increasing incidence, tuberculosis has become one of the leading causes of death among immunocompromised patients especially those who are HIV-TB coinfected. Kenya’s TB prevalence survey of 2015 indicated that the disease incidence was 233 per 100,000 (95% CI 188–266) [3] with the previous survey having been carried out in 1958. The results of the two surveys showed that the drivers of TB have certainly changed over the past 60 years. Another survey conducted in Kenya concluded that TB incidence has been previously underestimated [4].

**Fig. 1:**
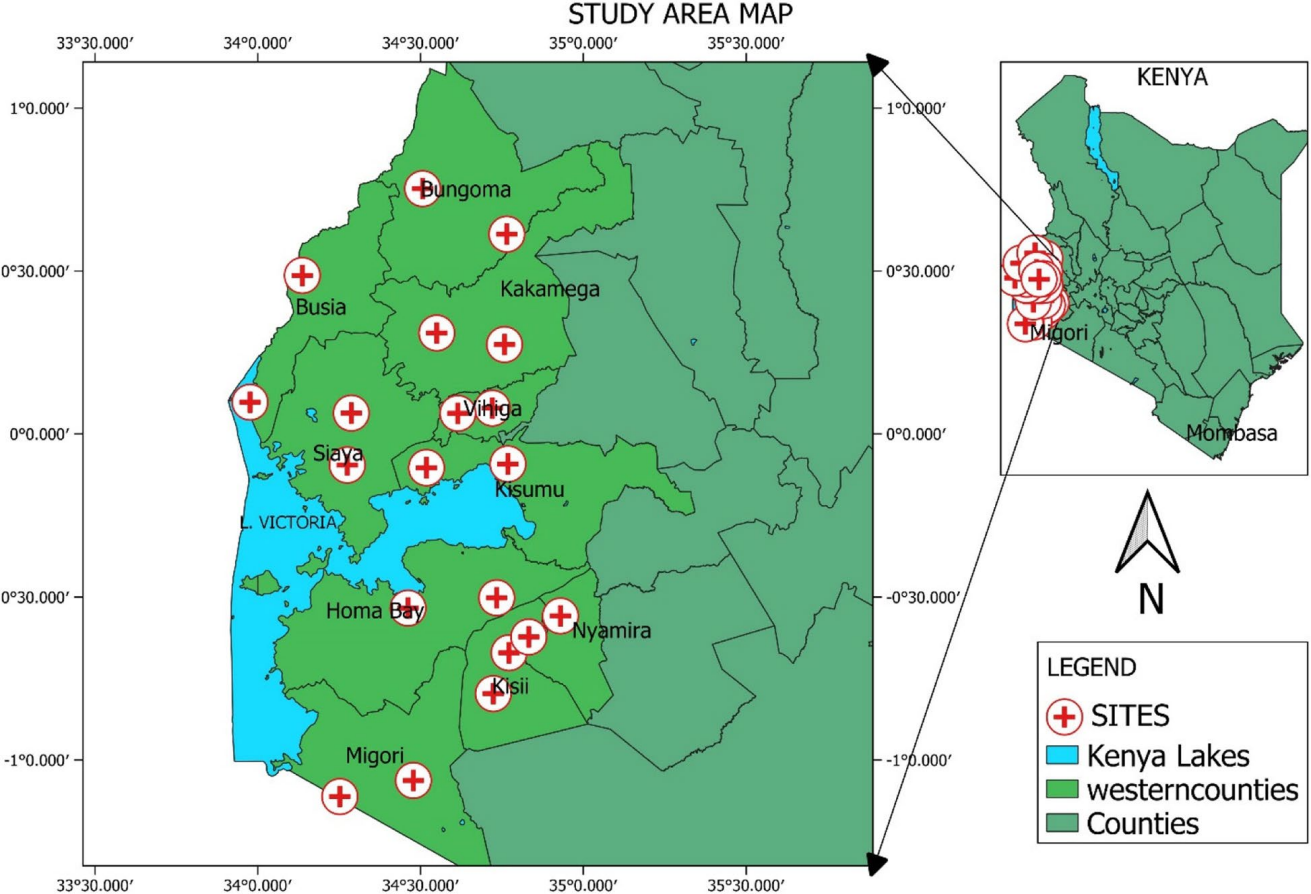
Map of Kenya showing study sites

Western Kenya consists of 10 county governments (Fig. 1) with varying number of sub-counties ranging from 5 to 12 depending on the population, landmass and vastness of the respective county. In that respect, Vihiga is the smallest county with 5 sub-counties while Kakamega is the largest with 12 sub-counties. Western Kenya counties border Tanzania and Uganda with close proximity to Rwanda, South Sudan and Somalia. Increasing interaction of citizens of these countries could be a risk factor to cross border transmission of tuberculosis further complicating the disease management in Kenya. This study established the population structure of *Mycobacterium tuberculosis* circulating in HIV negative and positive, and drug-resistant TB patients Western Kenya. The study also reports the existence of cross border transmission of tuberculosis. Evaluate the enablers, barriers and level of preparedness of western Kenya communities on tuberculosis cross border transmission.

Since 1940s when drug resistance was first reported, it has been identified as one of the major global challenge in the control and prevention of TB [5]. Tuberculosis burden in Africa is greatly driven by emergency and spread of Multidrug resistant (MDR) TB strains. While so many studies on molecular mechanisms of multidrug resistant and strain characterization have been carried out, early diagnosis remains a major set back of transmission of the strains due to increasing asymptomatic cases associated with MDR-TB [6]. Currently, drug susceptibility testing has been developed for diagnosis of MDR-TB, culture based phenotypic drug susceptibility has been relied on despite the fact that it takes long for results to be relayed. Delayed detection MDR-TB as a result of slow growth of the bacteria during culture and susceptibility test has been a challenge for early diagnosis and treatment of patients with MDR-TB [7]. Alternative methods for detection of MDR-TB such as the polymerase chain reaction-based methods which include molecular Line Probe Assay [8] has contributed greatly to early diagnosis despite the fact that the technology has not been rolled out in most low- and middle-income countries [9].

Molecular typing has led to great strides in genotypic characterization of *Mycobacterium tuberculosis* hence contributing significantly in the understanding of TB epidemiology. This has contributed immensely in improving TB control by availing critical information on transmission dynamics and determination of reactivation against exogenous reinfection [10, 11]. Genotyping of *M. tuberculosis* has been instrumental in disease TB surveillance with regard to investigating or confirming outbreaks, examining cross contamination that could have occurred in the laboratories and identification of the spread of successful clones like multidrug resistant TB. Genotypically, it’s possible to determine different population structures of *M. tuberculosis* thriving in a given geographical area with their possible pathological properties [12].

Spoligotyping technique has been used in *Mycobacterium tuberculosis* typing [13]. It is a PCR reverse-hybridization blotting based technique which has been widely used to study genetic diversity of the Direct Repeat locus of *M. tuberculosis* bacteria which is important in understanding the bacterium evolution, molecular epidemiology and population genetics. This technique is important in studying unique Events Polymorphism and insertion-Sequence-mediated genetic recombination [14]. This technique assesses the genetic diversity of direct repeat (DR) locus [15], which contains multiple 36-base pair (bp) DRs that are separated by 37 to 41 bp unique spacer sequences [16]. The 43 spacers are commonly used for genotyping [15]. Classical spoligotyping is performed by reverse line blot hybridization of biotinylated PCR products to a membrane with 43 covalently bound synthetic oligonucleotides representing the different spacers selected from *M. tuberculosis* H37Rv (spacers 1–19, 22–32, and 37–43) and *M. bovis* BCG (spacers 20–21 and 33–36) [17]. The presence and absence of each spacer is specific for each individual and used for genotyping.

Strain structure identification will help determining the impact of differing genetic characteristics among Mtb complex strains on vaccine efficacy, treatment outcome, development of MDR/XDR Mtb strains, and epidemiological outbreaks by focusing on the best-adapted human *M. tuberculosis* lineages. Strain structure will help understand factors contributing to *M. tuberculosis* success and survival strategies based on lineages and sub lineages survival characteristics. Cross border transmission of *M. tuberculosis* evaluation will help in form cross border events associated with it hence help in policy formulation of continuous surveillance and intervention at the border entry point. While determining enablers and barriers associated with cross boarder transmission prevention of *M. tuberculosis* is important in informing proper management and strategy for those identified to be carrying cross border strains.

## Materials and methods

### Study design and population

A cross sectional study design was applied in the study with multistage sampling method to select a single sub-county in each of the study counties. Smear positive tuberculosis patients above 18 years who had resided in the study area for at least 6 months were eligible for enrolment with only those who consented to the study recruited. Tuberculosis-HIV coinfected and non-coinfected were proportionately recruited based TB-HIV coinfection prevalence in each county as reported by Ministry of Health.

### Case definition

A case of tuberculosis disease was defined as a patient having two acid fast bacilli positive test or a single positive *Mycobacterium tuberculosis* culture. A cluster case was defined as any TB case from a study isolates whose strain type based on standard spoligotyping assay was not distinguishable from at least one other case. On the other hand, unique cases shared a unique strain not found in the study population.

### Sampling method

A multistage sampling method was used where a single sub-county was randomly selected followed by sampling two high volume health facility from each sub-county. Consenting study subjects with at least 2 smear positive sputum at the time of enrolment were randomly selected. A total of 553 samples were collected across 10 counties between 2018 and 2019. Sample size was calculated based on [18].

### Collection and transportation of sputum

Two sputum specimens (spot and early morning) were collected from participants with presumptive TB under the supervision of trained and competent medical staff. Upon consenting to the study, the patients were requested to cough so that expectoration came from deep down the chest as possible, and spit into a sterile 50 ml screw cap tubes. The samples were refrigerated at 4 °C awaiting transportation in cool boxes to the Mycobacteria Reference Laboratory, Moi University School of Medicine (MRL, MUSOM) weekly for analysis. Samples were processed within 7 days of collection in order to minimize loss of viability of the *Mycobacterium*. Participants also underwent phlebotomy for HIV testing. The blood was delivered into Vacutainer Brand STERILE interior EDTA (K3) tubes and stored at –20 °C waiting processing. The samples were transported in cool boxes to MRL, MUSOM, Eldoret, and processed within 2 weeks [19].

### Identification of *Mycobacterium* from clinical samples

BACTEC MGIT 960 assay was used in determining *Mycobacterium* strains from clinical samples. Briefly, the specimens ware decontaminated and digested with an equal volume of 4% sodium hydroxide for 15 minutes while vortexing after every 5 minutes. Decontaminated sputum was then transferred into a 50 ml corning tube, and then centrifuged at 3000 rpm for 15 minutes and the supernatant discarded. Phosphate-buffered saline (PBS, pH 6.8) was added to the residue raising the volume to 50 ml. The content was then centrifuged at 3000 rpm for 15 minutes and the supernatant discarded. 0.5 ml of PBS, pH 6.8 was added to resuspend the pellets, then 0.5 ml of resuspended deposit was added to the MGIT culture tube which were then incubated into the BACTEC MGIT 960 system until positivity was observed for positive samples or up to 8 weeks for negative samples [20]. Positive tubes were identified, removed and confirmed for acid fastness (AFB), for species identification and drug susceptibility testing and later storage of isolates.

### Identification *M. tuberculosis* from mycobacteria other than tuberculosis (MOTT) strains

The Capilla TB Test [21] was performed directly from positive cultures (from broth or from re-suspended colonies from solid media). Briefly, the Capilia TB assay was performed by placing 100 μl from a broth culture onto the specimen placement area of the Capilia TB cartridge, allowing a maximum of 15 min incubation and observing a purple–reddish color change in the test area. This was the indicator that the sample under test was *Mycobacterium tuberculosis* complex, and those that did not show the purple–reddish color was considered as MOTTS and therefore eliminated from the study.

### Culture

The ABBL MGIT tube (from Becton Dickinson) containing 7 mL modified middle brook 7H9 broth was used, to which an enrichment supplement as well as a mixture of antibiotics consisting of polymyxin B, amphotericin B, nalidixic acid, trimethoprim, and azlocillin were added [22]. After inoculation, the tubes were incubated at 37 °C. Readings were taken daily for the first 3 weeks and once a week thereafter for culture positivity until the end of 6 weeks using the BBL Micro MGIT system [23]. All the positive tubes were further confirmed by ZN staining and a sub culturing on blood agar plate and a LJ slant. The time to detection (TTD) of Mycobacteria were based on the date of the earliest instrumental indication of positivity.

### Extraction of *M. tuberculosis* DNA

Each sample pellet was dispersed in 10 mM Tris-1 mM EDTA buffer containing 0.1% Tween 80 and 2 mg/ml lysozyme. The tube was incubated for 2 hrs at 37 °C with intermittent shaking and centrifuged. The pellet was lysed by redissolving in TE buffer containing Proteinase K (100 μg/ml) and 1% (w/v) sodium dodecyl sulphate and incubated for 1 hr. at 37 °C. Then, an equal volume of TE saturated phenol: chloroform: iso-amyl alcohol (25:24:1 v/v/v) was added. The aqueous phase was transferred to another sterile tube and 0.1 volume of cold 3 M sodium acetate (pH 5.2) was added. The sample was then mixed by inversion and placed on ice for 10 min before centrifugation for 1 min at a speed of *11,000*×*g at room temperature*. The supernatant was transferred to another sterile tube and DNA precipitated by the adding acrylamide (20 μg/ml), 0.05 volumes of 3 M sodium acetate and 2.5 volume of ethanol. The DNA was then washed, dried, and dissolved in 25 μl sterile triple distilled water [24].

### Quantification of extracted *M. tuberculosis* DNA

Quantification of DNA was done spectrophotometrically by measuring absorbance at 260 nm for comparative quantitative analysis of DNA [25]. Serial dilutions of DNA extracted were prepared ranging from 0.5 μg to1.0 μg. All of these dilutions were amplified to determine the minimum amplifiable quantity of DNA.

### Spoligotyping

In this study, the classical 43-spacer format of spoligotyping was performed as previously described [16]. DNA samples of the *M. tuberculosis* H37Rv and *Mycobacterium bovis* BCG strains were included as positive controls. Molecular biology-grade water was used as negative control. The spoligotypes (presence and absence of spacers) were then recorded in 43-digit binary format and compared with those recorded in the SpolDB4 database (http://www.pasteur-guadeloupe.fr:8081/ SITVIT_ONLINE/) to identify the Spoligotype International Type (SIT) and family [14]. For the spoligotypes that matched the SITs, but could not be related to any family (i.e., unknown), and for the spoligotypes that were not present in the SpolDB4 database (e.g., orphan), the SPOTCLUST program, which were built from the spolDB3 database (http://tbinsight.cs.rpi.edu/run_spotclust.html) [26], was used to search for *M. tuberculosis* family similarity. In the SPOTCLUST analyses, the family assignation was retained when the probability is ≥90%. Nevertheless, the final designation of families and subfamilies was also based on the MIRU-VNTR data.

### Spoligotype cluster analysis

Association between patient characteristics and genotype clustering were evaluated using logistic regression and frequency analysis. The potential risk factors for clustering included age, HIV status, sex, level of education, occupation, monthly income, size of the house, cigarette smoking, alcohol use and cross boarder travels. Bivariate analysis was performed to determine the association of potential risk factors with genotypic clustering followed by multivariate logistic regression adjusting for potential cofounders. Since TB cases were from adjacent and approximate counties and that all cases were notified within the same calendar year, the specific geographical and temporal timing of cases were not used in the definition of a cluster. Lineage clustering rate was computed from the difference between the total strains clustered cases in each lineage and the number of clusters divided by the sum of cases in the MTB lineage.

## Results

### Social demographic characteristics of the study population (*N* = 553)

Participants in this study were 18 years and above with the most affected being between 25 and 30 years representing 33.1% of the total sample size. 48.3% of the participants were HIV positive while 51.7% were HIV negative. Majority of the participants were males (62.4%) while the rest were females. Most of the participants (44%) were primary school leavers, 26% with informal education, 23% secondary school leavers while participants with tertiary education were 7%. Self-employed participants 39%, those with informal employment were 23.6%, unemployed were 16.6% while 15% were employed with 1.8% still in school. Participants with monthly income in the bracket of 10,000–19,999 were 31.5% with the least being those with monthly income of Ksh. 50,000 and above (Table 1).

**Table 1:** Social demographic Characteristics of the study population (*N* = 553)

Variable	Status	Frequency	Percentage
**Age**	18–24	145	26.1
	25–30	183	33.1
	31–36	107	19.3
	37–42	47	8.5
	43–48	35	6.3
	49–54	18	3.3
	55–60	12	2.2
	61+	6	1.1
**HIV status**	Positive	267	48.3
	Negative	286	51.7
**Sex**	Female	208	37.6
	Male	345	62.4
**Education**	No formal	144	26
	Primary	243	44
	Secondary	127	23
	Tertiary	39	7
**Occupation**	Employed	83	15
	Self employed	218	39
	Informal employment	128	23
	Unemployed	103	16.6
	Student	10	1.8
	Other	11	2
**Monthly income**	Less than Ksh. 1000	105	19
	Ksh. 1000–9, 999	160	28.9
	Ksh. 10, 000–19, 999	174	31.5
	Ksh. 20,000–29, 999	48	8.7
	Ksh. 30,000-39,999	34	6.1
	Ksh. 40,000-49,999	18	3.3
	Ksh. 50,000 +	14	2.5
**Size of the house**	Single room	122	22.1
	Bedsitter	11	2
	One bedroom	207	37.4
	Two bedroom	191	34.5
	Other	22	4
**Cigarate smocking**	Yes	132	23.9
	No	421	76.1
**Alcohol use**	Yes	151	27.3
	No	402	72.7
**Cross boarder travels**	Yes	211	38.2
	No	342	61.8

In terms of housing, 37.4% of the participants were living in single bedroomed houses, 34.5% in two bedroomed houses, 2% in bedsitters, 22.1% in single rooms, the rest were 2%. Also, among the participants, 76.1% were non cigarette smokers while 23.9% were smokers. On the same note, 72.7% did not take alcohol while 27.3% were alcohol users.

In terms of local and international travels, 38.2% were involved in cross boarder travel while 61.8% did not (Table 1).

### Spoligotyping

The distribution of *M. tuberculosis* strains in Western Kenya is highly diversified with Euro American strains (H, LAM, T, S and X), Indo Oceanic (MANU), East Asia strains and Beijing strains being identified across the 10 counties of Western Kenya. Lineage distribution pattern revealed that the most predominant lineage was CAS 220 (39.8%) followed by Beijing 128 (23.1%). The other lineages identified were T, LAM, H, X, S and MANU which were quantified as 87(15.7%), 67(12.1%), 16(2.8%), 10(1.8%), 8(1.4%) and 5(0.9%) respectively (Table 2, Fig. 1).

**Table 2:**
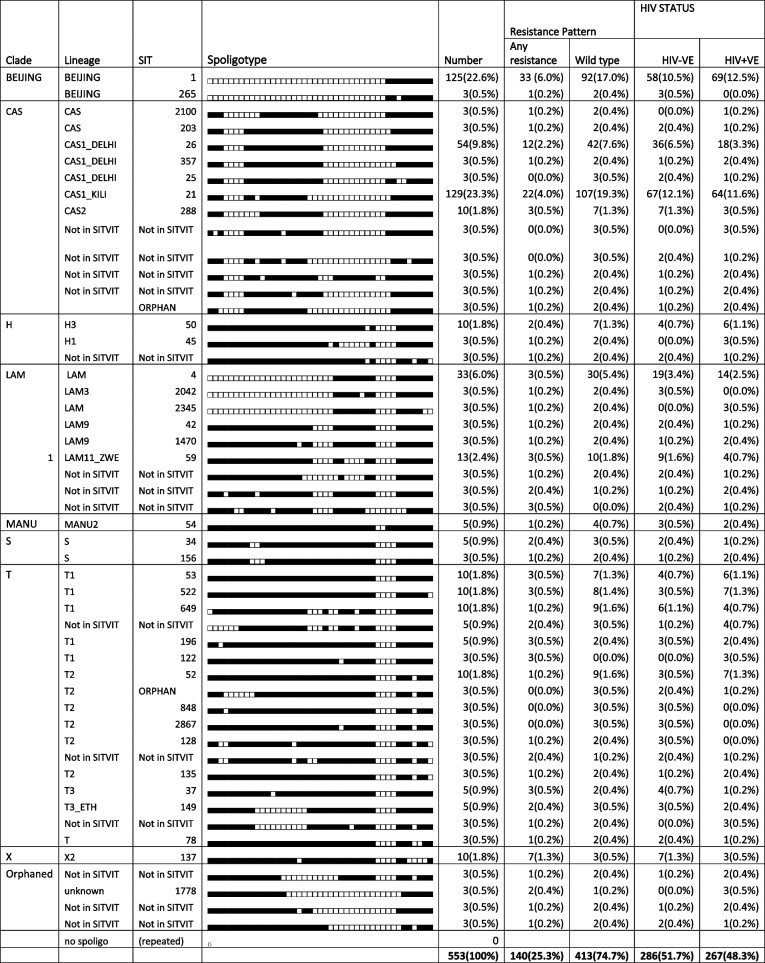
Showing *M. tuberculosis* strain distribution pattern as compared to SITVIT database. Strain distribution in HIV positive and negative TB patients and also their distribution patterns in TB drug resistant and drug sensitive patients

Clade distribution among HIV negative and positive, CAS and Beijing strains were the most equally distributed among HIV positive and negative participants. T and unknown strains were predominantly detected mostly in HIV positive respondents. While CAS, H, LAM, MANU, S, T and X were predominant in HIV negative (Table 2, Fig. 1).

Clade distribution among wild type and resistant *M. tuberculosis* TB patients, revealed that Beijing lineage was most identified in resistant strains than wild type strains with orphaned strains also being detected (Table 2, Fig. 1).

**Fig. 2:**
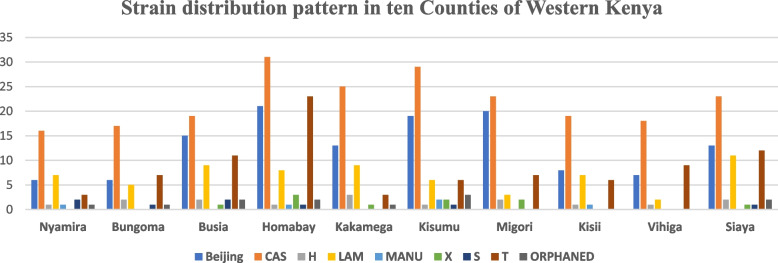
Clade distribution in 10 counties of Western Kenya

Across all the 10 counties of Western Kenya, CAS strain was the most predominant followed by Beijing, T and finally LAM (Fig. 2). In Bungoma county, Beijing, CAS, H, LAM and T *M. tuberculosis* lineages were detected representing 6 (1.1%), 17 (3.1%), 2 (0.4%), 5 (0.9%) and 7 (1.2%) respectively (Table 3).

**Table 3:** Strain distribution pattern in Western Kenya counties

	Beijing	CAS	H	LAM	MANU	X	S	T	ORPHANED
**Nyamira**	6(1.1%)	16(2.9%)	1(0.2%)	7(1.3%)	1(0.2%)	0(0.0%)	2(0.4%)	3(0.5%)	1(0.2%)
**Bungoma**	6(1.1%)	17(3.1%)	2(0.4%)	5(0.9%)	0(0.0%)	0(0.0%)	1(0.2%)	7(1.2%)	1(0.2%)
**Busia**	15(2.7%)	19(3.4%)	2(0.4%)	9(1.6%)	0(0.0%)	1(0.2%)	2(0.4%)	11(2.0%)	2(0.4%)
**Homabay**	21(3.8%)	31(5.6%)	1(0.2%)	8(1.4%)	1(0.2%)	3(0.5%)	1(0.2%)	23(4.2%)	2(0.4%)
**Kakamega**	13(2.4%)	25(4.5%)	3(0.5%)	9(1.6%)	0(0.0%)	1(0.2%)	0(0.0%)	3(0.5%)	1(0.2%)
**Kisumu**	19(3.4%)	29(5.2%)	1(0.2%)	6(1.1%)	2(0.4%)	2(0.4%)	1(0.2%)	6(1.1%)	3(0.5%)
**Migori**	20(3.6%)	23(4.1%)	2(0.4%)	3(0.5%)	0(0.0%)	2(0.4%)	0(0.0%)	7(1.3%)	0(0.0%)
**Kisii**	8(1.4%)	19(3.4%)	1(0.2%)	7(1.2%)	1(0.2%)	0(0.0%)	0(0.0%)	6(1.1%)	0(0.0%)
**Vihiga**	7(1.3%)	18(3.3%)	1(0.2%)	2(0.4%)	0(0.0%)	0(0.0%)	0(0.0%)	9(1.6%)	0(0.0%)
**Siaya**	13(2.3%)	23(4.1%)	2(0.4%)	11(2.0%)	0(0.0%)	1(0.2%)	1(0.2%)	12(2.2%)	2(0.4%)
**TOTAL**	**128 (23.1%)**	**220(39.8%)**	**16(2.8%)**	**67(12.1%)**	**5(0.9%)**	**10(1.8%)**	**8(1.4%)**	**87(15.7%)**	**12(2.2%)**

In Busia, four major lineages were detected with CAS being the dominant strain with a proportion of 20 (3.4%), followed by Beijing (2.7%), T (2.0%) and LAM (1.6%). Subsequently, two unknown strains of *M. tuberculosis* were detected. Similarly, the most prevalent lineage detected in Homa Bay was CAS (8.1%), followed by Beijing (4.9%) with 0.5% of the total samples typed were unknown (Table 3).

In Kakamega, Beijing, CAS, H, LAM, T and X lineages were detected at the proportion of 2.4, 4.5, 0.2, 0.5, 1.6, 0.5 and 0.2% respectively. Subsequently, in Kisumu county just like in Kakamega County CAS lineage was the most prevalent at a proportion of 5.2%, followed by Beijing and LAM both with the proportion of 3.4 and 1.1%, H had a proportion of 0.2%, T and X having a proportion of 1.1 and 0.4% respectively. The proportion of unknown lineage in Kisumu County was 0.5% (Table 3).

In Migori 0.7% of the samples typed were unknown and did not belong to any lineage were detected, however the majority of strains identified belonged to CAS lineage (4.1%) followed by T lineage (1.3%). Beijing lineage identified in Migori were 3.6% in proportion to the total sample typed while LAM, H and X were represented by 0.5, 0.4 and 0.4% respectively (Table 3).

In Kisii, 0.3% were unknown strains not belonging to any lineage as compared to SITVIT database. In this county, majority of samples typed belonged to Beijing strain followed by CAS, LAM, T, H and MANU at the proportion of 3.4, 0.2, 1.1, 0.2 and 0.2% respectively (Table 3).

In Vihiga, the predominant clades were CAS, Beijing and T just as many other Western Counties represented by a proportion of 3.3, 2.3 and 1.6% respectively. On the other hand, in Siaya County, four lineages were found to dominate among TB patients; CAS, Beijing, LAM and T at the proportion of 4.1, 2.3, 2.0% and then 2.0%. Then Finally, Nyamira recorded CAS at a proportion 2.9%, LAM had a proportion 1.3% and finally Beijing 1.1% (Table 3).

Bivariate analysis of potential risk factors and genotypic clustering of *M. tuberculosis* showed that six factors showed significant association. These factors included age of 25–30 with odd ratio of 0.95, confidence interval of 0.35–2.27 and *P*-value of 0.003. HIV status was significantly associated with genotypic clustering with OR (Odd Ratio) of 1.73, CI (Confidence Interval) of 0.42–5.21 and *P* value of 0.04. Those with monthly income of between Ksh. 1000-Ksh. 9999 were significantly associated with genotypic clustering having OR of 1.28, CI of 0.62–4.00 and *P*-value of 0.046. There was significant association between those living in single rooms with genotypic clustering with OR of 1.43, CI of 0.46–4.31 and P-value of 0.049. Alcohol and cross boarder travels were also significantly associated with genetic clustering with OR of 0.87, 0.93; CI of 0.34–5.34, 0.46–3.92 having *P*-values of 0.003, 0.002 respectively. In multivariate logistic regression adjusting for potential cofounders three potential risk factors were significant. HIV status (OR = 1.52, CI = 0.29–3.68 and P value of 0.001), Alcohol use (OR = 0.59, CI = 0.43–3.12 and P-value =0.001) and cross border travel (OR = 0.61, CI = 0.49–3.87 and P value = 0.026) (Table 4).

**Table 4:** Bivariate and Multivariate risk factor association with genotypic patterns of *M. tuberculosis* strain isolates

Variable	Status	TOTAL	Genotypic pattern		Bivariate Analysis			Multivariate Analysis		
			Unique	Clustered	Unadjusted			Adjusted		
					OR	CI(95%)	*P*-Value	OR	CI (95%)	*P*-Value
**Age**	18–24	145 (26.22)	14(2.53)	131(23.69)	Ref					
	25–30	183(33.09)	23(4.15)	160(28.93)	0.95	0.35–2.27	0.003**	0.63	0.26–2.32	0.056
	31–36	107(19.35)	17(3.07)	90(16.27)	2.13	0.96–6.12	0.81	1.67	0.33–3.23	0.72
	37–42	47(8.50)	3(0.54)	44(7.96)	1.93	041–4.61	0.94	0.72	0.21–1.89	0.65
	43–48	35(6.32)	3(0.54)	32(5.79)	2.16	0.98–6.78	0.31	1.53	0.43–2.87	1.45
	49–54	18(3.25)	4(0.72)	14(2.53)	1.34	0.35–3.72	0.13	1.21	0.27–2.67	1.20
	55–60	1(0.18)	1(0.18)	11(1.99)	0.85	0.23–3.10	0.12	0.45	0.12–2.98	0.35
	61+	6(1.08)	0(0.00)	6(1.08)	1.23	0.16–3.81	0.48	0.94	0.43–2.78	0.87
**HIV status**	Positive	267(48.28)	26(4.70)	241(43.58)	1.73	0.42–5.21	0.04**	1.52	0.29–3.68	0.001**
	Negative	286(51.71)	17(3.07)	269(48.64)	Ref					
**Sex**	Female	208(37.61)	13(2.35)	195(35.26)	1.67	0.23–3.02	0.27	1.32	0.23–3.78	0.97
	Male	345(62.38)	17(3.07)	326(58.95)	Ref					
**Education**	No formal	144(26.04)	18(3.25)	126(22.78)	2.61	0.87–5.92	0.92	2.21	0.49–3.98	1.34
	Primary	243(43.94)	14(2.53)	229(41.41)	Ref					
	Secondary	127(22.97)	19(3.43)	108(19.52)	2.32	0.61–5.24	0.71	2.13	0.49–3.09	1.98
	Tertiary	39(7.05)	4(0.72)	35(6.32)	3.61	1.02–7.31	0.21	3.32	0.67–5.76	2.34
**Occupation**	Employed	83(15.01)	15(2.71)	68(2.30)	1.67	0.24–3.06	0.36	1.43	0.45–2.97	1.23
	Self employed	218(39.42)	15(2.71)	203(36.71)	Ref	Ref				
	Informal employment	128(23.15)	21(3.80)	107(19.35)	1.52	0.21–2.98	0.54	1.22	0.22–3.21	0.98
	Unemployed	103(18.62)	13(2.35)	90(16.27)	1.43	0.19–2.2.61	0.25	1.31	0.57–2.61	1.13
	Student	10(1.81)	2(0.36)	8(1.45)	0.98	0.37–3.12	0.46	0.69	0.22–2.22	0.34
	Other	11(1.99)	1(0.18)	10(1.80)	1.73	0.31–2.98	0.41	1.57	0.23–2.01	0.87
**Monthly income**	Less than Ksh. 1000	105(18.98)	10(1.80)	95(17.18)	Ref					
	Ksh. 1000–9, 999	160(28.93)	11(1.99)	149(26.94)	1.28	0.63–4.00	0.046**	1.19	0.27–3.02	0.52
	Ksh. 10, 000–19, 999	174(31.46)	17(3.07)	157(28.75)	1.73	0.58–4.32	0.82	1.53	0.21–2.56	0.98
	Ksh. 20,000–29, 999	48(8.68)	4(0.72)	44(7.96)	1.82	0.69–3.92	0.76	1.67	0.36–2.63	1.98
	Ksh. 30,000-39,999	34(6.15)	2(0.36)	32(5.79)	1.23	0.42–3.62	0.34	0.98	0.32–3.21	0.71
	Ksh. 40,000-49,999	18(3.25)	1(0.18)	17(3.07)	1.68	0.41–4.31	0.36	1.42	0.51–2.21	1.34
	Ksh. 50,000 +	14(2.53)	2(0.36)	12(2.17)	1.53	0.29–3.93	0.54	1.37	0.31–2.87	1.43
**Size of the house**	Single room	122(22.06)	17(3.07)	105(18.99)	1.43	0.46–4.31	0.049**	1.27	0.29–3.02	0.056
	Bedsitter	11(1.99)	3(0.54)	8(1.45)	1.83	0.32–3.61	0.71	1.59	0.36–3.42	0.78
	One bedroom	207(37.43)	21(3.80)	186(33.63)	Ref					
	Two bedroom	191(34.54)	18(3.25)	173(31.28)	1.92	0.67–5.42	0.83	1.63	0.41–3.67	0.73
	Other	22(3.98)	2(0.36)	20(3.62)	1.86	0.20–5.31	0.47	1.53	0.37–3.45	0.56
**Cigarate smocking**	Yes	132(23.87)	7(1.26)	125(22.6)	1.81	0.43–4.82	0.41	1.51	0.46–3.32	1.21
	No	421(76.13)	14(2.53)	407(73.60)	Ref					
**Alcohol use**	Yes	151(27.31)	16(2.89)	135(24.41)	0.87	0.34–5.34	0.003**	0.59	0.43–3.12	0.001**
	No	402(72.69)	11(1.99)	391(70.70)	Ref					
**Cross boarder travels**	Yes	211(38.15)	16(2.89)	195(35.26)	0.93	0.46–3.92	0.002**	0.61	0.49–3.87	0.026**
	No	342(61.84)	17(3.07)	325(58.77)	Ref					

^**^P-value of <0.05 was considered significant. *CI* Confidence interval

## Discussion

Strain structure identification is key in understanding the circulating *M. tuberculosis* clades within TB patients hence help in informing targeted policy formulation with regards to enhancing preventive and control strategies. This was the first study to determine strain diversity and distribution in Western Kenya circulating among HIV negative and positive patients in the region. HIV/AIDS is the major risk factor to acquiring TB in Western Kenya due to its effecting compromising human immune system [27]. high prevalence rates of the HIV epidemic in this region especially among the five counties; Kisumu, Homa Bay, Siaya, Busia and Migori [28]. The High prevalence rate has been contributed by socio culture behavior of the natives of this area [29]. It is therefore important to understand the *M. tuberculosis* strain structure circulating in clinical isolates of this region. Additionally, there has been increased circulation of drug resistant *M. tuberculosis* of varying degrees ranging from mono resistant, multi resistant and poly resistant strains. Drug resistance structure of *M. tuberculosis* under study was reported by Ogwang et al., [30] thus this study intended to establish *M. tuberculosis* drug resistance strain structure through spacer oligonucleotide typing (Spoligotyping). Our findings indicate a circulation of diverse strains of *M. tuberculosis* in the study area. The study established that despite the existing of predominant strains like CAS, Beijing and LAM, there was no clear association with either HIV status of the patient or drug resistant *M. tuberculosis*.

The distribution of *M. tuberculosis* strains in Western Kenya is highly diversified with Euro American strains (H, LAM, T, S AND X), Indo oceanic (MANU) and East African strains being identified in addition to Beijing strain. Studies by Merker et al., [31] showed that Africa is the only continent with all the seven human adapted lineages with further evidence indicating that MTBC originated from Africa and spread globally through human migration events and slave trade [32]. This could explain the findings of this study with regards to genetic diversity of *M. tuberculosis* lineages in Western Kenya.

Spoligotyping patterns of some clinical isolates could not be matched to other reported in international database hence referred to us orphaned. This unique strain needs further analysis using other molecular epidemiology tools including Mycobacterium Interspersed Repetitive Units (MIRU) or Variable Number of Tandem Repeats (VNTR) to shed more insights on their epidemiology. These orphaned strains could indicate either recent or sporadic TB infections in Western Kenya.

The detection of different lineages of *M. tuberculosis* in Western Kenya maybe an indicator of global diversity of *M. tuberculosis* strains with different origin given that there are several counties under study that are bordering neighboring countries like Uganda and Tanzania where most of these strains has been reported [33–35]. The findings of this study is in agreement with previous studies which indicated that CAS is wide spread and predominant in Kenya, Uganda, Tanzania and Ethiopia [36] which are East African Community countries which allow for free movement of its citizen across the border.

The discovery of wide spread Beijing strain which has been reported as the most lethal driver of drug resistance and reinfection [37] could be the contributing factor to persistent of the disease in Western Kenya. The Beijing strain has been reported as the most virulent strain globally [38]. This has been because it’s the most favored strain with mutations hence easily develops drug resistant [39].

On the other hand, despite Western Kenya Counties bearing the largest burden of HIV pandemic, there was no evidence that specific strains have an association of HIV even though the status is a risk factor to acquiring tuberculosis due to suppressed immune system. Despite other studies associating TB drug resistance to Beijing lineage, this study did not find any correlation between the strain and resistance. Therefore, resistance in this case could be associated to poor drug adherence, failure in prompt diagnosis, migration, poverty and increased TB/HIV cross infection [30].

Most of the *M. tuberculosis* isolates from the study population were clustered which may inform that a large proportion of were recent infection. Further, this could indicate that the clustered strain might be having a higher transmissibility of higher chances off progressing into disease stage immediately after transmission. There was also a sizable number of unique strains, indicating the main drivers of persistent tuberculosis in the population that undergoes latent stage before reactivation occurs. This could be the main public health change in terms of control and prevention of tuberculosis in the population. Latency affects early diagnosis and therefore management of tuberculosis.

Significant association in bivariate analysis especially age, monthly income and size of houses used by patients could be as a result of potential cofounders, this could further be explained in multivariate logistic regression adjusting for potential cofounders which showed that the above factors were not significant. Multivariate logistic regression analysis showed HIV status, alcohol use and cross border travels as having significant association with genetic clustering of tuberculosis. HIV virus is known to fight the immune system hence exposing populations to opportunistic diseases like tuberculosis. Compromised immunity could accelerate disease progression to active tuberculosis immediately after infection. Alcohol is a social drink taken bay revelers who share cups, syphons and other drinking utensils. Western Kenya is one of the leading traditional, crude and other forms of alcohol users [40] further alcohol use have been reported to increase the burden of tuberculosis [41]. Local and international travels have also been reported to increase the risk of tuberculosis transmission [42]. Emigrants globally have been reported to contribute to global distribution of tuberculosis. This has resulted to genetic diversity of *M. tuberculosis* strains globally [43].

## Conclusion

There exist diverse strains of *M. tuberculosis* across the 10 counties of Western Kenya. Predominant distribution of clustered genotype as opposed to unique points due to the fact that most TB cases in this region are as a result of resent transmission other than activation of latent TB.

### Recommendation

The identification of diverse *Mycobacterium tuberculosis* in 10 counties of Western Kenya calls for aggressive control and prevention strategies to curb mortalities associated with pulmonary tuberculosis. Spoligotyping patterns of some clinical isolates could not be matched to others reported in international database hence referred to us orphaned need to be further characterized using other molecular epidemiology tools including Mycobacterium Interspersed Repetitive Units (MIRU) or Variable Number of Tandem Repeats (VNTR) to shed more insights on their epidemiology. These orphaned strains could indicate either recent or sporadic TB infections hence the need for aggressive intervention.

### Strength and limitation of the study

The study has demonstrated the existence of genetic diversity of *M. tuberculosis* circulating in Western Kenya population. Further we have demonstrated the existence of fast progressing pathogens to full blown disease stage and those that undergo latency through M tuberculosis cluster analysis. Additionally, the study has identified potential risk factors that are likely to be the drivers of tuberculosis in Western Kenya. However, we were unable to further characterize the orphaned strains through other available genotypic characterization tools to strengthen our findings.

## Data Availability

The data that support the findings of this study are available from Division of Tuberculosis, Leprosy and Lung Diseases, Ministry of Health and Research Division Jomo Kenyatta University of Agriculture and Technology, Kenya but restrictions apply to the availability of these data, which were used under license for the current study, and so are not publicly available. Data are however available from the authors upon reasonable request and with permission of the Head of Division Tuberculosis, Leprosy and Lung Diseases Kenya P.O. Box 20781–00202 Nairobi, Kenya. Tel: +(254) 773 977 440, Jomo Kenyatta University of Agriculture and Technology P.O. Box 62000–00200, Nairobi, Telephone + 254–67-5870000/1/2/3/4/5 and Corresponding author through email: macogwang2012@gmail.com.
